# Epidemiological evolution of gingival cancer in Spain from 2001 to 2022: a longitudinal study

**DOI:** 10.4317/medoral.27465

**Published:** 2025-08-16

**Authors:** Jorge Celis-Dooner, María Cristina Mateo-Sidrón-Antón, Rocío Cerero-Lapiedra, Luis Alberto Moreno-López

**Affiliations:** 1 DDS, MSc. Complutense University of Madrid, Madrid, Spain; 2 DDS, MSc, PhD. Titular professor, Complutense University of Madrid, Madrid, Spain; 3 DDS, MSc, PhD. Adjunct professor, Complutense University of Madrid, Madrid, Spain

## Abstract

**Background:**

Over the past decades, the literature has described epidemiological changes in oral cancer. However, few updated studies specifically address this issue, particularly those examining it separately from pharyngeal cancer. Some authors suggest gingival cancer is the only intraoral cancer with a higher prevalence among women.

**Material and Methods:**

A retrospective longitudinal study was conducted using the Specialized Care Activity Registry from the Minimum Basic Data Set to obtain data on gingival cancer patients in Spain from 2001 to 2022.

**Results:**

Data from 5,749 patients showed 51.8% were men and 48.2% women. A final predominance in women was observed. The average recorded age was 68.3 years, increasing in both sexes over time. A low frequency of tobacco and alcohol consumption was noted. The age-adjusted incidence was 0.61, with a convergence between sexes due to increased female incidence.

**Conclusions:**

During the study period, a reversal in the male-to-female ratio was observed, along with an increase in recorded age, a preference for the mandible over the maxilla, low tobacco and alcohol consumption, and incidence convergence between sexes due to rising female cases.

** Key words:**Oral cancer, gingival cancer, spain, epidemiology.

## Introduction

Oral Cancer (OC) can be understood as all malignant neoplasms that originate within the boundaries of the oral cavity, including the lips, intraoral anatomical structures, and major and minor salivary glands. Oral Squamous Cell Carcinoma (OSCC) accounts for approximately 90% of the malignant neoplasms diagnosed in this area (1).

OC ranks sixteenth in global incidence, with a total of 389,846 new cases diagnosed in 2022 (2). It is estimated that by 2045, the annual incidence will rise to 579,096 new cases worldwide (3). Regarding mortality, the five-year survival rate after diagnosis is approximately 50% (4). Globally, oral cancer caused a total of 188,438 deaths in 2022 (2), representing 1.9% of all cancer-related deaths, and the number of deaths is expected to increase to 292,865 by 2045 (3).

Patients diagnosed with OC are usually over 40 years old, with an average age of around 60 years. It more frequently affects men than women, with a ratio of 1.5:1 (5). In recent decades, changes in these epidemiological variables have been observed, including an increase in cases, a decrease in the age of onset, a rise in female patients, and the emergence of cases in individuals without known risk factors. This shift has become an active area of research within the scientific community (6).

Gingival cancer accounts for 10% to 15% of OC cases and is typically ranked as the third or fourth most common anatomical location within this group. This relative frequency varies depending on the studied population, sometimes reaching the second position (7,8). Some authors report that gingival cancer is more common in female patients, which would distinguish it from other OC locations. However, this has not been universally confirmed by the literature (9).

The aim of this study is to describe the epidemiological evolution of gingival cancer in the Spanish population from 2001 to 2022.

## Material and Methods

- Desing

A retrospective longitudinal study was conducted between January and June 2024, using the databases of the Minimum Basic Data Set - Hospitalization (CMBD-H) for data from 2001 to 2015 and the Specialized Care Registry (RAE-CMBD) for data from 2016 to 2022. Both databases constitute the most comprehensive data source available in Spain regarding patients. Between 2015 and 2016, a modification in the structure of these databases led to a change in their name. This change involved adjustments in the data collection methodology, which may have affected the frequency of the data between the two periods. Additionally, the version of the International Classification of Diseases (ICD) was updated from the 9th to the 10th edition, both for diagnoses and procedures. The databases collect information on all hospital admissions within the network of Spanish public hospitals and a non-quantified portion of private hospitals. They are publicly available and can be accessed through the Specialized Care Information System of the Ministry of Health of Spain. The data is anonymized to protect the identity of patients and hospitals.

-Selection of patients and study variables

Data was requested for all patients registered with a diagnosis of oral and oropharyngeal cancer as the cause of admission between 2001 and 2022. From these, patients over 18 years of age with gingival cancer as the primary diagnosis (admission cause) or those who were admitted for another pathology but were diagnosed with gingival cancer (secondary diagnosis) were selected. The following variables were collected: year of registration, sex, age at admission, lesion location according to the ICD-9 or ICD-10 versions, and smoking and alcohol consumption habits, according to the relevant coding in both editions of the ICD. For the joint analysis of data from both ICD versions, the eCIEmaps tool, provided by the Ministry of Health in its version v4.0.06, was used.

- Calculation of incidence

The age-adjusted incidence was calculated using the Spanish population data obtained from the Spanish National Institute of Statistics (INE) as the susceptible population and the reference standard population proposed by the European Union's statistical office (Eurostat).

- Statistical analysis

The Shapiro-Wilk normality test was used for the ratio between men and women by year, the incidence for the overall population, and for men and women by year. The Kolmogorov-Smirnov test was used for the age variable. To compare the mean age of patients by sex, the Mann-Whitney U test was applied. Linear regression was used to assess the time evolution of the ratio between men and women, global incidence, and incidence in men and women, with time in calendar years as the independent variable. Jamovi software (Version 2.3.28.0) was used, and a *p-value* of less than 0.05 was considered statistically significant.

## Results

Data was collected for 100,869 patients with a primary diagnosis of oropharyngeal cancer, of which 69,206 were cases of intraoral cancer, and 5,749 were cases of gingival cancer, accounting for 8.31% of all intraoral cancer cases.

- Sex

In gingival cancer, 2,978 cases (51.8%) were male patients, while 2,771 cases (48.2%) were female patients.

**Figure 1:**
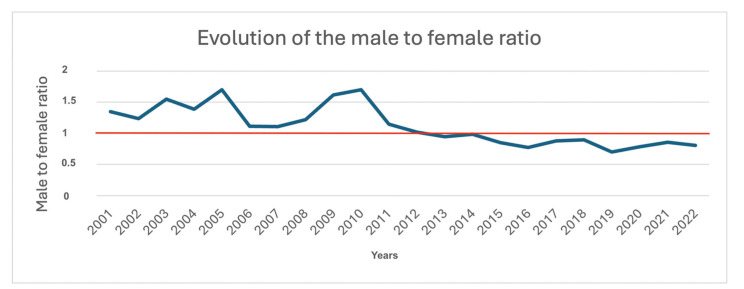
Evolution of the male to famale ratio.

During the study period, a noTable epidemiological shift occurred in the male-to-female (M/F) ratio. From 2001 to 2012, a male predominance was observed, with the ratio fluctuating between 1.7:1 and 1.02:1. However, in 2013, this trend reversed, with female predominance emerging for the first time, reflected in a ratio of 0.95:1. This female predominance persisted throughout the study period, reaching its lowest value of 0.70:1 in 2019 (Fig. 1).

- Age

**Figure 2:**
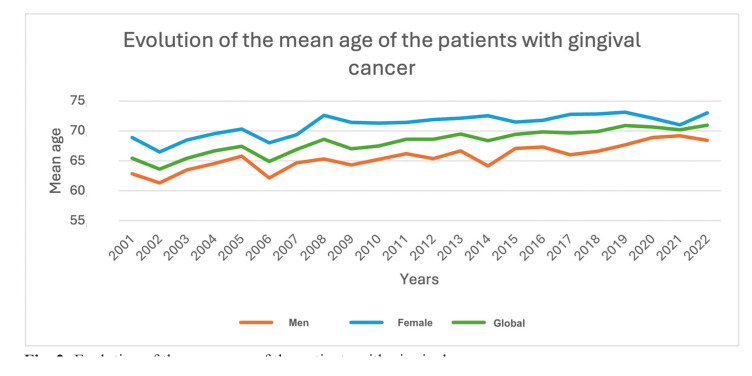
Evolution of the mean age of the patients with gingival cancer.

The overall mean age was 68.3 years (SD ± 13), with male patients having a mean age of 65.55 years (SD ± 12.73) and female patients a mean age of 71.26 years (SD ± 12.66). Over the course of the study, a clear trend emerged, showing an increase in the average age of gingival cancer cases, both for the overall population and for male and female patients separately. Additionally, female patients consistently had a higher mean age than male patients throughout the entire study period (Fig. 2).

Statistical analysis revealed that male patients were diagnosed with gum cancer an average of 5.8 years earlier than female patients (*p*<0.001).

- Location

It was observed that 1,277 (22.21%) cases of gingival cancer developed in the upper gum, 2,738 (47.63%) in the lower gum, and 1,734 (30.16%) did not specify the location. A global ratio of lower gum to upper gum location of 2.14:1 was observed, with a range between 1.39:1 and 4.8:1.

- Tabacco consumption

**Figure 3:**
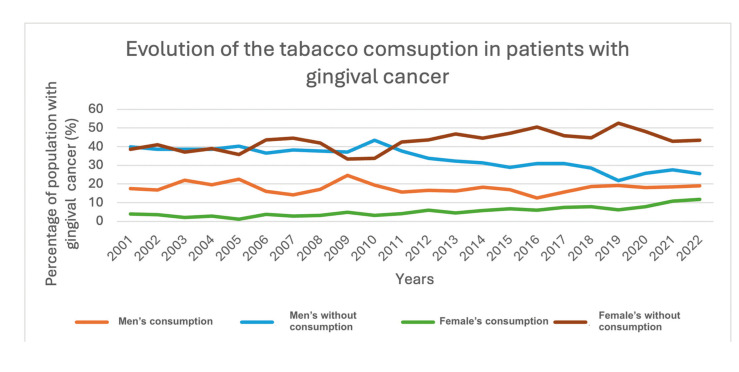
Evolution of the tabacco consumption in patients with gingival cancer.

Regarding of Tabacco consumption, it was observed that female patients had a lower frequency of smoking compared to male patients. In both sexes, there was a percentage increase in smokers with gingival cancer, with a greater increase in female patients. However, it is noteworthy that most of the population with gingival cancer were non-smokers (Fig. 3).

- Alcohol consumption

Regarding problematic alcohol consumption by sex, a low prevalence was observed in both men and women, with it being more common in men, ranging from 1.51% to 8.02% in men and 0% to

2.31% in women, respectively.

- Age-adjusted incidence

The age-adjusted incidence per 100,000 inhabitants was 0.61 for the overall population, 0.7 for male patients, and 0.53 for female patients throughout the study period.

Regarding the evolution of incidence in the total population, a minimum incidence of 0.51 and a maximum of 0.81 patients per 100,000 inhabitants were observed, with a clear increasing trend from 2001 to 2015. In 2016, a sharp decline in incidence was noted, recording 0.47 patients per 100,000 inhabitants, which represented a 41.25% decrease compared to the previous year.

**Figure 4:**
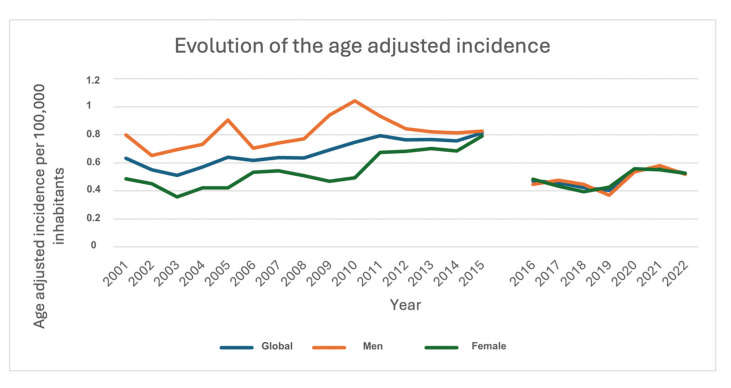
Evolution of the age adjusted incidence.

In terms of incidence by sex, it was observed that between 2001 and 2015, the incidence in male patients was higher than in female patients. In 2001, the incidence was 0.8 patients per 100,000 inhabitants, reaching its peak of 1.04 patients per 100,000 inhabitants in 2010. For female patients, there was an upward trend, starting with an incidence of 0.49 patients per 100,000 inhabitants in 2001, reaching a maximum of 0.79 patients per 100,000 inhabitants in 2015. In that year, the overall incidence, as well as the incidence in males and females, converged within a narrow margin, which remained sTable until the end of the study (Fig. 4). incidence, as well as the incidence in males and females, converged within a narrow margin, which remained sTable until the end of the study (Fig. 4).

Regarding the model obtained through linear regression, it was observed that the overall incidence (R²: 0.057, p: 0.282) and the incidence in females (R²: 0.065, p: 0.251) did not show a significant relationship with time, indicating that time was not a significant predictor for the variation in these incidences. However, a significant negative relationship was found between the incidence in males and time (R²: 0.294; p: 0.009).

## Discussion

The main objective of this study was to describe the epidemiological evolution of gingival cancer in the Spanish population from 2001 to 2022. One of the most relevant findings is the consistent decrease in the male-to-female ratio, which reversed in 2013, showing, for the first time, a higher number of female patients compared to male patients. This trend continued throughout the study period. While global literature has reported an epidemiological shift over the past two decades, marked by an increase in the incidence of oral cancer and a decline in the male-to-female ratio (1), this is the first time that a nationwide epidemiological study has recorded a higher incidence in females, with this trend persisting and even intensifying for over 10 years. It is important to note that this finding pertains specifically to gingival cancer and does not necessarily represent the overall distribution of oral cancer in Spain. However, these results seem to align with those described in the study by Capote-Moreno *et al*., which assessed the epidemiological evolution of oral cancer in a Madrid hospital over 39 years. Their study reported a male-to-female ratio of 4.6:1 in the 1980s, which gradually decreased over time, reaching a ratio of 1.1:1 by the 2010s (10).

While the current epidemiological shift in oral cancer could be explained by changes in the frequency of risk factor exposure, it is tempting to hypothesize that proliferative verrucous leukoplakia may be influencing these results. This pathology, first described by Hansen *et al*. in 1985 (11), is characterized by a higher prevalence in women, affecting the gingiva, and has a high progression rate to carcinoma (60% to 100% of cases) (12). Although this explanation may not fully account for the male-to-female ratio shift observed in this study, it provides an interesting angle for further investigation. Another possible explanation relates to the characteristics of the epithelium in the affected area. Rautava *et al*. describe how neoplasms originating in keratinized epithelium tend to be more common in females (8).

Regarding tobacco and alcohol consumption as risk factors for the development of oral cancer, a low frequency of consumption was observed, with tobacco use being more common in male patients. In female patients, there was a slight trend toward an increasing percentage of smokers with cancer, although this never exceeded 12% of the total population. Conversely, the percentage of female patients without a history of smoking also increased, reaching a maximum of 45% of the total population. These findings seem paradoxical, as the study shows an increase in female cases compared to male cases, which, at first glance, does not appear to correlate with tobacco use as a risk factor. This observation is rare in the literature, as smoking is generally described as the primary risk factor for oral cancer, and in gum cancer epidemiological studies, smoking is reported in 50% to 62% of cases (13,14). This could support the hypothesis that proliferative verrucous leukoplakia is closely related to this epidemiological shift, as studies indicate that this diagnosis is not heavily influenced by tobacco use (15). It is also important to note that the coding of risk factors in the CMBD may be subject to variations in coders at each hospital and the updates in their definitions, which can explain some of the noticeable changes between 2015 and 2016, along with the possibility of underreporting by hospitals.

Regarding age-adjusted incidence, the analysis should be limited to trends over the study period and the male-to-female relationship due to the coding changes noted between 2015 and 2016. An increase in the incidence among females was observed, leading to convergence with male patients. In 2016, a decline in this incidence was noted, likely due to the change from ICD-9 to ICD-10, where certain case definitions were modified. However, the convergence between sexes persisted. A similar trend was observed in a study by Stepan *et al*. (16) in the United States, where a decrease in male cases and an increase in female cases was noted. By 2017, the last year of their study, the incidence approached 0.4 patients per 100,000 inhabitants, which aligns closely with the findings in this study for the same year.

-Limitations

The CMBD database is a hospital activity database, so it is not focused on the calculation and recording of epidemiological and clinical variables. As a result, these variables may be over- or under-recorded. There is an indeterminate number of patients treated in the private system, whose data is not included in this database, meaning that incidence data may be underestimated. Lastly, the change in coding from ICD-9 to ICD-10 has resulted in modifications to how variables are organized in the database, which has impacted the reporting, as described in the incidence data. This change should be interpreted with caution, particularly in the long-term series. Nevertheless, the CMBD database remains a valuable tool for epidemiological studies, as it has been used by other researchers to publish data, due to its large sample size, which provides high representativeness and statistical power (17).

## Conclusions

The epidemiological evolution of gingival cancer in Spain from 2001 to 2022 reveals a series of changes. There was a predominance of male patients from 2001 to 2012, followed by a shift in 2013, when more cases were recorded in female patients, a trend that continued until 2022. During the study period, an increase in the mean age at diagnosis was observed, along with a low frequency of tobacco use and alcohol consumption. Lastly, a convergence in incidence between males and females was noted, driven by an increase in the incidence among female patients, which remained consistent through 2022.

It should be considered that these changes in the epidemiology of gingival cancer may reflect shifts in the disease's risk factors and/or changes in the biological behavior of the gingival tissue. Furthermore, they highlight the need to adapt educational, healthcare, and public health management resources in response to the changes detected through epidemiological research within the population.
